# An Excellent Clinical and Radiological Response Pattern to Pembrolizumab in a Patient With Metastatic Adrenocortical Carcinoma and Lynch Syndrome

**DOI:** 10.1002/iju5.70040

**Published:** 2025-05-26

**Authors:** Yuki Shimozawa, Yosuke Yasuda, Emiko Sugawara, Ryosuke Oki, Kosuke Takemura, Tetsuya Urasaki, Ryo Fujiwara, Noboru Numao, Junji Yonese, Takeshi Yuasa

**Affiliations:** ^1^ Department of Genitourinary Oncology, Cancer Institute Hospital Japanese Foundation for Cancer Research Tokyo Japan; ^2^ Department of Pathology, Cancer Institute Hospital Japanese Foundation for Cancer Research Tokyo Japan; ^3^ Department of Medical Oncology, Cancer Institute Hospital Japanese Foundation for Cancer Research Tokyo Japan

**Keywords:** immune checkpoint inhibitor, Lynch syndrome, metastatic adrenocortical carcinoma, mismatch repair genes, pembrolizumab

## Abstract

**Introduction:**

The prognosis of unresectable metastatic adrenocortical carcinoma is very poor. We report a case of Lynch syndrome accompanying metastatic adrenocortical carcinoma treated with pembrolizumab.

**Case Presentation:**

A 73‐year‐old woman was diagnosed with left adrenocortical carcinoma and multiple lung, liver, and lymph node metastases. First‐line mitotane therapy failed due to toxicity and progressive disease. Immunohistochemical analysis of mismatch repair proteins revealed an MSH6 deficiency. Pembrolizumab monotherapy was started for microsatellite instability‐high/mismatch repair–deficient malignant disease. After the first administration, we experienced temporal clinical findings considered to reflect the collapse of tumors. She gained remarkable reductions in all lesions after four cycles. Genetic analysis disclosed the germline pathogenic variant of *MSH6*, so this case was diagnosed as Lynch syndrome.

**Conclusion:**

We report a patient with metastatic adrenocortical carcinoma in Lynch syndrome who demonstrated an excellent response to pembrolizumab. Genetic analyses can play a beneficial role in cases of adrenocortical carcinoma.

**Summary**

- We report a case of Lynch syndrome accompanying metastatic adrenocortical carcinoma treated with pembrolizumab after mitotane monotherapy that demonstrated an excellent response to pembrolizumab.
- Genetic analyses can provide useful information for definitive diagnosis and may play a beneficial role for both the patient and their relatives.

**Abbreviations**
^18^F‐FDG: fluorodeoxyglucose F18
ACC: adrenocortical carcinoma
CT: computed tomography
DHEA‐S: dehydroepiandrosterone sulfate
dMMR: mismatch repair–deficient
EDP: etoposide, doxorubicin, and cisplatin
MMR: mismatch repair
MSI‐H: microsatellite instability‐high
PET: positron emission tomography

## 1 Introduction

ACC is a rare malignancy whose estimated incidence is approximately 1–2 per a million population per year [1]. Surgery is the most effective therapy for not only local disease but also oligometastatic disease. The prognosis of unresectable metastatic ACC is poor, and the 5‐year overall survival rate is 0%–17% for stage IV cancers [2]. Recently, due to its expected efficacy and manageable safety profile, pembrolizumab monotherapy was approved for MSI‐H/dMMR solid tumors [3]. Here, we report a case of a patient with left ACC and multiple lung, liver, and lymph node metastases. She experienced an excellent radiological response after four cycles of pembrolizumab therapy.

## 2 Case Presentation

**FIGURE 1:**
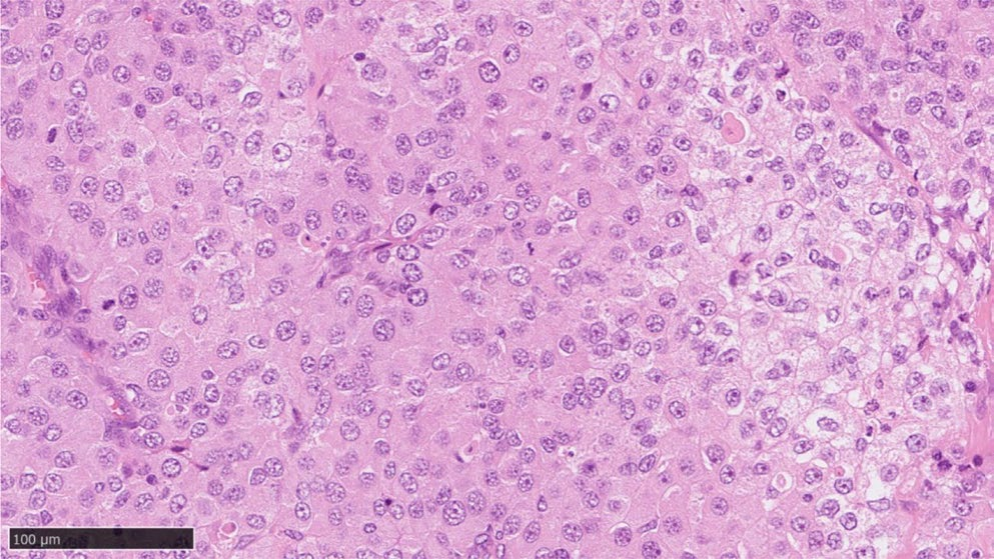
Histopathological appearance of the metastatic lymph node.

**FIGURE 2:**
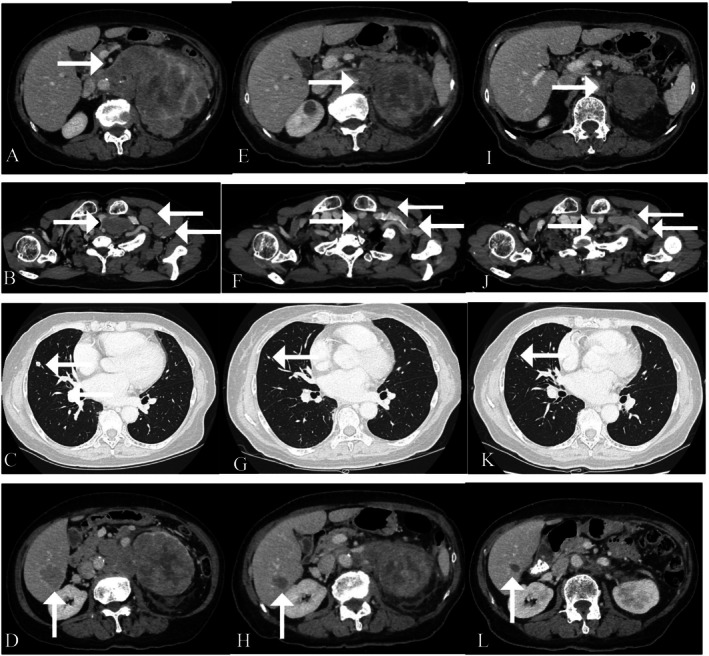
Radiological presentation of metastatic adrenocortical carcinoma. Before the introduction of pembrolizumab therapy (left panels), the first time that tumor shrinkage was found (middle panels) and after cycle 4 pembrolizumab (right panels) for primary lesions (A, E, I), cervical lymph nodes (B, F, J), lung (C, G, K), and liver (D, H, L) metastases.

A 73‐year‐old Japanese female patient presented with left cervical lymphadenopathy and left lumbar pain. Thoraco‐abdominal CT scans demonstrated a huge adrenal mass (11.4 cm in diameter), multiple cervical, mediastinal, and retroperitoneal lymphadenopathies, and multiple hepatic and lung metastases. Strong ^18^F‐FDG accumulation was confirmed in all lesions using PET/CT scanning. Her DHEA‐S level was high, and her adrenocorticotropic hormone level was mildly low despite the normal cortisol level. She underwent a left cervical lymph node biopsy. Pathological analyses disclosed metastatic ACC (Figure 1). Surgical treatment was contraindicated in this case due to the multiple metastatic regions. Systemic treatment with mitotane (1500 mg on Day 1, 3000 mg on Day 2, and 4500 mg from Day 3/body) was, therefore, started 1 month after her first visit. However, mitotane therapy was discontinued on Day 29 due to hepatic dysfunction (G2). At the same time, CT scans demonstrated progressive disease (Figure 2A–D).

**FIGURE 3:**
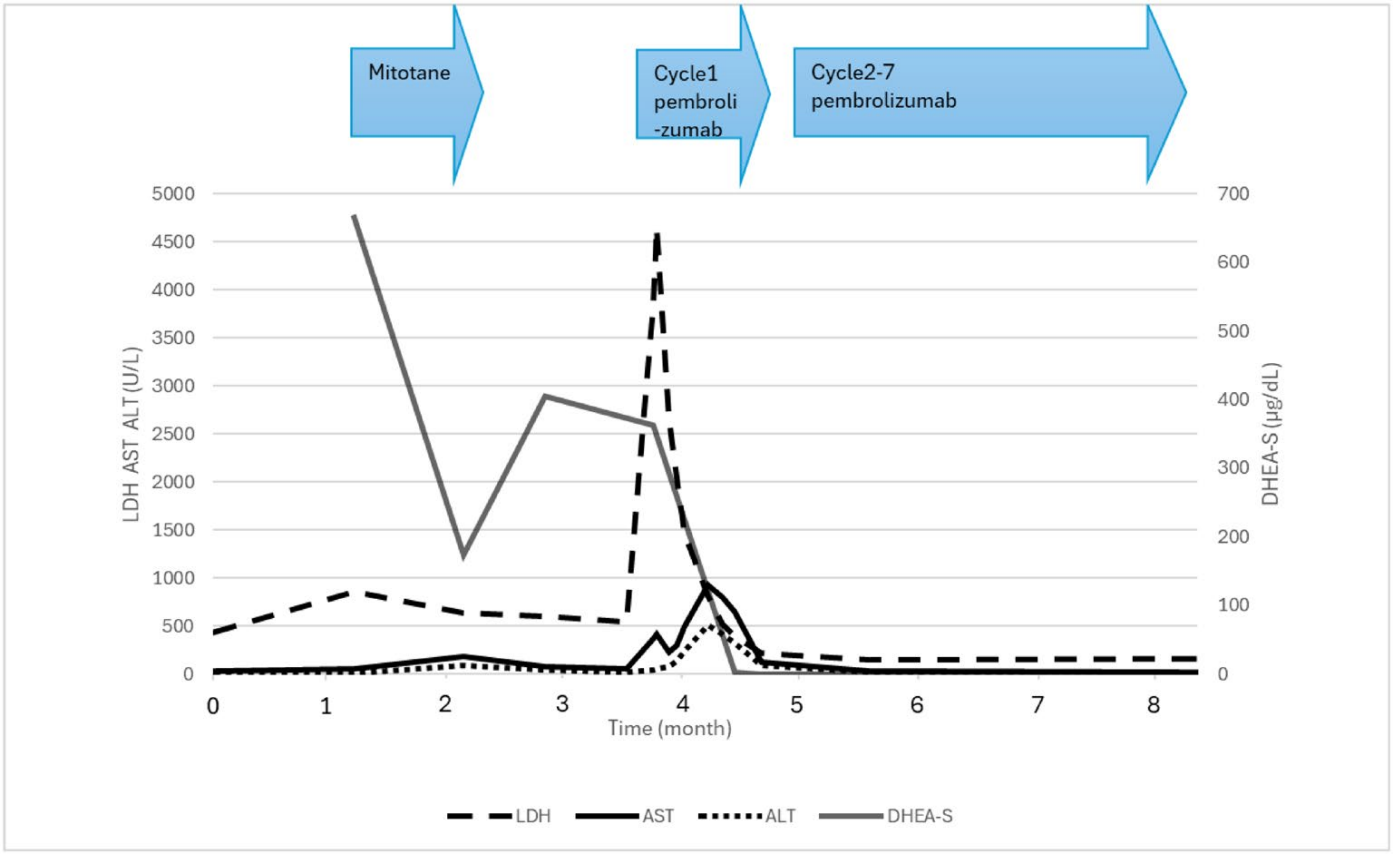
Schematic presentation for medical therapy and serum variables for advanced adrenocortical carcinoma. ALT, alanine aminotransferase; AST, aspartate aminotransferase; DHEA‐S, dehydroepiandrosterone sulfate; LDH, lactate dehydrogenase.

Immunohistochemical analysis of MMR proteins revealed retained MLH1, PMS2, and MSH2, whereas an MSH6 deficiency was disclosed. Therefore, the ACC was diagnosed as an MSI‐H/dMMR malignant disease. Pembrolizumab (200 mg/body every 3 weeks) monotherapy was started 3 months after her first visit. Immediately after the first administration, the patient developed a high fever (> 39.0°C) and mildly impaired consciousness. She also had laboratory abnormalities, including notable increases in lactate dehydrogenase and C‐reactive protein levels, so she was temporarily hospitalized. Although thoraco‐abdominal CT scans were performed, no infectious lesion was disclosed. The patient's symptoms improved over the next few days, and the indicated abnormalities also peaked out. Her transaminase levels increased with a delay, but those also peaked out. We performed CT scans again when her transaminase levels increased. Although there was no hepatic abnormal lesion, we found tumor shrinkage (Figure 2E–H). Therefore, we considered that these clinical symptoms, including a transient increase in serum enzymes, were not from immune‐related adverse events, but reflected the quick breaking down of the malignant tumors. Consequently, we re‐started pembrolizumab therapy. After the fourth cycle, her latest CT scans demonstrated remarkable reductions in all lesions. The adrenal mass was especially reduced to 5 cm (Figure 2I–L). At the same time, next‐generation sequencing analysis (Foundation One) identified mutations in the following genes: *MSH6*, *CBL*, *DNMT3A*, *NF1*, *PTEN*, *RAD51D*, *SDHB*, *SMARCB1*, and *TP53*. DNA change of c.2665C>T and protein change of p.Q889* were disclosed on *MSH6*. The tumor mutational burden was 31.4 mutations/Mb. Although the patient had no familial history suggestive of Lynch syndrome, she had a history of uterine endometrial cancer and had undergone total hysterectomy at the age of 59. Subsequent genetic analysis disclosed a germline pathogenic variant of *MSH6* and, therefore, she was genetically diagnosed with Lynch syndrome. Figure 3 describes the schematic presentation for medical therapy and serum variables for advanced ACC. Her DHEA‐S level decreased with pembrolizumab therapy.

## 3 Discussion

Metastatic ACC is an aggressive cancer originating from the cortex of the adrenal gland and has a poor prognosis. Mitotane, which is an analog of the insecticide dichlorodiphenyltrichloroethane, is the only approved agent for unresectable and/or metastatic ACC. Although its precise mechanism of action is still unknown, mitotane is considered to change the peripheral metabolism of steroids and suppress adrenal steroidogenesis [4]. However, mitotane has low efficacy and a narrow therapeutic index and can thus cause serious side effects [4].

Various clinical trials have been and are being conducted. Among them, the EDP–mitotane trial for ACC was the largest (*n* = 304) in this rare disease [4]. In the randomly assigned open‐label clinical trial, patients treated with EDP–mitotane had a significantly higher response rate than those treated with streptozocin–mitotane (23.2% vs. 9.2%, *p* < 0.001) [4]. However, there was no significant difference in overall survival between the groups (14.8 and 12.0 months, *p* = 0.07), and the trial failed to accomplish its primary end point [4]. Relatively high serious adverse events (58.2%) were seen in patients treated with EDP–mitotane [4]. Therefore, EDP–mitotane has not been considered an established therapy for metastatic ACC.

One of the other candidate agents is an immune checkpoint inhibitor. In Japan, as companion diagnostics for pembrolizumab/MSI‐high, several diagnostics have been approved. Among them, we use the IHC method to determine MHC status using VENTANA OptiView Kits (manufactured by Roche Diagnostics) because VENTANA OptiView Kits can be performed in our institution and may have a shorter turnaround time than outsourcing. One phase II clinical trial of pembrolizumab therapy for advanced ACC has been conducted (*n* = 39). The trial's objective response rate and disease control rates were found to be 23% (9/38) and 52% (16/31), respectively [5]. Interestingly, six of the 38 patients (16%) had MSI‐H/dMMR tumors, which are more common in ACC than has been recognized [5]. The median overall survival rate in this pilot study was 24.9 months [5]. Consequently, various immune checkpoint inhibitor‐associated clinical studies are ongoing [6].

The patient described here had a germline mutation of *MSH6*. Lynch syndrome is one of the most common hereditary cancer syndromes. It is caused by a pathogenic germline variant in one of the MMR genes, which include *MHL1*, *MSH2*, *MSH6*, *PMS2*, and *EPCAM* [7, 8, 9]. Lynch syndrome is associated with the most common type of hereditary colorectal cancer, followed by uterine endometrial and urothelial cancers. In addition, the prevalence of Lynch syndrome among patients with ACC is 3.2%, which is comparable with the prevalence of Lynch syndrome in colorectal and uterine endometrial cancer [10]. Therefore, ACC is considered to be a Lynch syndrome‐associated cancer [9].

In conclusion, in this case study, we report a patient with metastatic ACC in Lynch syndrome who demonstrated an excellent clinical and radiological response pattern to immune checkpoint inhibitor therapy. As MSI‐H/dMMR tumors are more common in ACC (16%), genetic analyses of patients must be undertaken, as these can provide useful information for definitive diagnosis and also be beneficial for both the patient and their relatives.

## Ethics Statement

The authors have nothing to report.

## Consent

Informed consent from the patient was obtained.

## Conflicts of Interest

The authors declare no conflicts of interest.

## References

[1] B. C. James , B. Aschebrook‐Kilfoy , N. Cipriani , E. L. Kaplan , P. Angelos , and R. H. Grogan , “The Incidence and Survival of Rare Cancers of the Thyroid, Parathyroid, Adrenal, and Pancreas,” Annals of Surgical Oncology 23 (2016): 424–433, 10.1245/s10434-015-4901-9. 26467460

[2] R. Libé , “Adrenocortical Carcinoma (ACC): Diagnosis, Prognosis, and Treatment,” Frontiers in Cell and Development Biology 3 (2015): 45, 10.3389/fcell.2015.00045. PMC4490795 26191527

[3] L. Marcus , S. J. Lemery , P. Keegan , and R. Pazdur , “FDA Approval Summary: Pembrolizumab for the Treatment of Microsatellite Instability‐High Solid Tumors,” Clinical Cancer Research 25, no. 13 (2019): 3753–3758, 10.1158/1078-0432.CCR-18-4070. 30787022

[4] M. Fassnacht , M. Terzolo , B. Allolio , et al., “Combination Chemotherapy in Advanced Adrenocortical Carcinoma,” New England Journal of Medicine 366, no. 23 (2012): 2189–2197, 10.1056/NEJMoa1200966. 22551107

[5] N. Raj , Y. Zheng , V. Kelly , et al., “PD‐1 Blockade in Advanced Adrenocortical Carcinoma,” Journal of Clinical Oncology 38, no. 1 (2020): 71–80, 10.1200/JCO.19.01586. 31644329 PMC7351334

[6] D. Chukkalore , K. MacDougall , V. Master , M. A. Bilen , and B. Nazha , “Adrenocortical Carcinomas: Molecular Pathogenesis, Treatment Options, and Emerging Immunotherapy and Targeted Therapy Approaches,” Oncologist 29, no. 9 (2024): 738–746, 10.1093/oncolo/oyae029. 38381694 PMC11379653

[7] X. Li , G. Liu , and W. Wu , “Recent Advances in Lynch Syndrome,” Experimental Hematology & Oncology 10, no. 1 (2021): 37, 10.1186/s40164-021-00231-4. 34118983 PMC8199357

[8] D. T. Le , J. N. Uram , H. Wang , et al., “PD‐1 Blockade in Tumors With Mismatch‐Repair Deficiency,” New England Journal of Medicine 372, no. 26 (2015): 2509–2520, 10.1056/NEJMoa1500596. 26028255 PMC4481136

[9] R. Oki , T. Urasaki , A. Ueki , et al., “A Radiological Complete Response to Pembrolizumab in a Patient With Metastatic Upper Urinary Tract Urothelial Cancer and Lynch Syndrome,” International Journal of Urology: Case Reports 6, no. 28 (2022): 33–36, 10.1002/iju5.12542. 36605683 PMC9807341

[10] V. M. Raymond , J. N. Everett , L. V. Furtado , et al., “Adrenocortical Carcinoma Is a Lynch Syndrome‐Associated Cancer,” Journal of Clinical Oncology 31, no. 24 (2013): 3012–3018, 10.1200/JCO.2012.48.0988. 23752102 PMC3739861

